# Polidocanol Sclerotherapy in Hereditary Hemorrhagic Telangiectasia Patients Does Not Seem to Decrease Epistaxis‐Related Outpatient and Emergency Visits—A Retrospective View

**DOI:** 10.1111/coa.70059

**Published:** 2025-11-12

**Authors:** Elsa‐Leea Kotola, Johanna Wikstén, Eeva Castrén

**Affiliations:** ^1^ Faculty of Medicine University of Helsinki Helsinki Finland; ^2^ Department of Otorhinolaryngology and Head and Neck Surgery Helsinki University Hospital Helsinki Finland

**Summary**

- Hereditary hemorrhagic telangiectasia (HHT) is a rare inherited disease characterised by severe epistaxis.
- Polidocanol sclerotherapy for HHT‐related epistaxis has previously been shown to reduce epistaxis and improve quality‐of‐life.
- The impact of polidocanol sclerotherapy for HHT‐related epistaxis on the hospital resources is unknown.
- We studied how polidocanol sclerotherapy affects the number of epistaxis‐related outpatient and emergency visits in HHT‐patients.
- Despite the previously reported positive effects of polidocanol sclerotherapy, we observed no change in epistaxis‐related hospital visits in HHT‐patients.

## 1 Introduction

Hereditary hemorrhagic telangiectasia (HHT) is a rare inherited disease characterised by the presence of telangiectasia in the nasal and gastric mucosa and arteriovenous malformations (AVM) in the lungs, brain, and liver [1]. All known gene defects underlying the disease are related to the endothelial transforming growth factor beta family. These mutations in HHT patients lead to unregulated vessel wall regeneration in mucous membranes and internal organs leading to activation of angiogenesis. This results in dilated and tortuous postcapillary veins that eventually join the dilated arteries via capillaries. Subsequently, the capillary segments disappear and direct arteriovenous connections are formed [2, 3].

The diagnosis of HHT is based on the Curaco criteria, which include recurrent epistaxis, telangiectasias of the skin and mucosa, visceral AVMs and a positive family history for HHT [4]. Troublesome epistaxis is the most common symptom of HHT impairing the most the patients' quality of life. The nosebleeds are caused by ruptured intranasal telangiectasia, which can lead to frequent emergency room visits, hospital stays, anaemia and blood transfusions. Symptoms often aggravate with age [5].

Different treatments have been applied to control HHT‐related epistaxis and to improve the patients' quality of life. No curative treatment for HHT exists. The options to control epistaxis are topical agents, systemic medical treatment or locally invasive procedures on the nasal mucosa. Traditional procedures include cauterization, laser, septodermoplasty, or even nasal closure. Arterial embolization has also been used [6]. More invasive procedures require an operating room (OR), as do severe bleedings, which may unfortunately occur outside of office hours.

Currently, sclerotherapy has shown promising results to control epistaxis in HHT patients [7, 8]. Polidocanol, also known as lauromacrogol 400 or aethoxysklerol is the most used sclerosing agent. Polidocanol is stable at room temperature and can be applied under local anaesthesia [8]. This treatment technique to control epistaxis in HHT patients was first reported by Ramírez et al. in 2000 [9], followed by a larger study by Morais et al. in 2012 [8]. Polidocanol sclerotherapy has been shown to relieve the frequency and severity of epistaxis, reduce anaemia and blood transfusions, and improve the quality of life. Serious sclerotherapy complications, such as unilateral blindness are extremely rare [7, 8].

However, the impact of sclerotherapy for HHT patients on the number of hospital visits is not known. Our objective was to study whether polidocanol sclerotherapy influences the number of epistaxis‐related emergency room and outpatient visits, and on the need for emergency OR procedures.

## 2 Methods

We conducted a database search from our institution's electronic patient registry using ICD‐10 code for HHT (I78.0) and utilised our hospital's registry for rare diseases from 2003 to 2021, to identify all HHT patients in our catchment area. Demographic and HHT‐related data were collected. We selected those HHT patients who had received polidocanol sclerotherapy at our Otorhinolaryngology department and had a follow‐up period of a minimum of 3 years before and 1 year after the year the sclerotherapy was initiated. For these patients, all emergency, outpatient, and OR visits as well as all procedures to control epistaxis in our unit were registered. Due to the retrospective study setting, the quality of life or the Epistaxis Severity Score (ESS) data was not sufficiently available. The data was analysed using the SPSS statistical package for social sciences, 28.0 version. The study protocol was accepted by the local ethics committee.

## 3 Results

**FIGURE 1:**
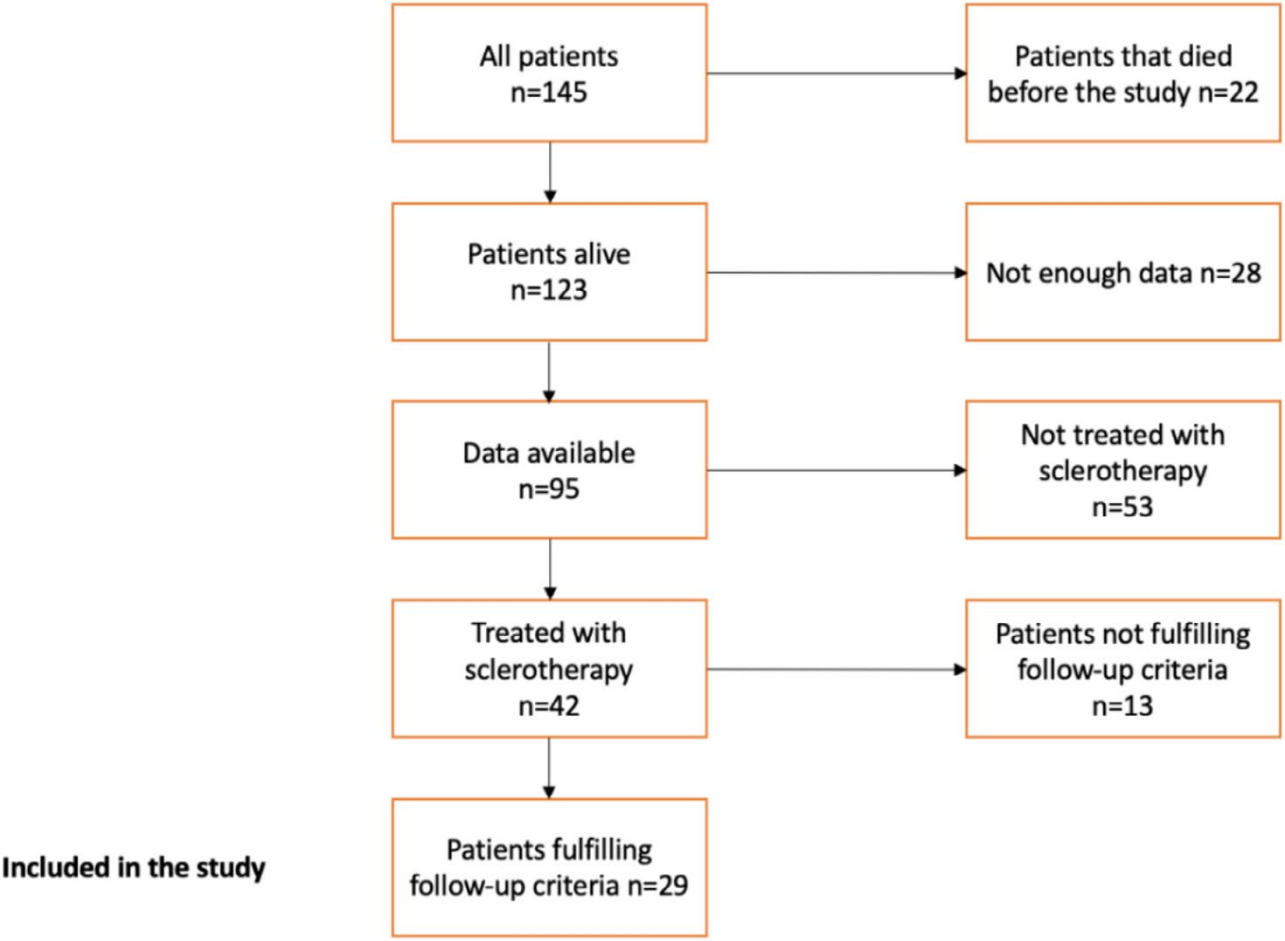
Flow chart of the patient selection.

Our study cohort consisted of a total of 145 HHT patients; 116 were excluded from the study according to the inclusion criteria presented in Figure 1. Patients excluded because not enough available data were mainly treated outside our hospital district. Finally, the study included 29 patients. The demographic and HHT‐related information of the selected patients is presented in Table 1. The mean age was 65 years (range 28–90 years). There were on average 30 injection visits per year. Patients received on average 7.45 sclerotherapy sessions (range 1–35). The total number of polidocanol sclerotherapies during the study period was 216.

**TABLE 1:** Demographic and HHT‐related data.

Demographic data	*N* total = 29 (%)
Gender	
Male	11 (38%)
Female	18 (62%)
Age (years)	
20–39	3 (10%)
40–59	6 (21%)
60–79	17 (59%)
80+	3 (10%)
Gene mutation	
ACVRL1	10 (34%)
ENG	3 (10%)
Unknown/not tested	16 (55%)
HHT organ manifestations	
Epistaxis	29 (100%)
Pulmonary AVM	12 (41%)
Brain AVM	0 (0%)
Liver AVM	5 (17%)
Gastrointestinal telangiectasia	8 (26%)
Skin and mucosal telangiectasia	20 (69%)

Abbreviations: AVM = arteriovenous malformation, HHT = hereditary hemorrhagic telangiectasia.

### 3.1 Outpatient and Emergency Department Visits

The mean annual number of outpatient visits per patient before the initiation of polidocanol sclerotherapy was 1.30, and 1.64 respectively during the post‐treatment period. After the initiation of sclerotherapy, 10 patients experienced a decrease in outpatient visits, while eight patients experienced an increase. However, the overall number of visits increased. Statistically, the result was insignificant (*p* = 0.192).

Before the initiation of polidocanol sclerotherapy, the mean annual number of emergency department visits per patient was 0.21, and 0.32 respectively during the post‐treatment period. The highest mean number of visits per year for a single patient was 1.33 in the pre‐treatment period, and respectively 2.67 during the post‐treatment period. In total, eight patients had emergency department visits during the pre‐treatment period. Emergency department visits decreased in four patients and increased in eight patients. Seventeen patients showed no change in the number of emergency department visits. Overall, emergency department visits increased during the post‐treatment period. However, the numbers were statistically insignificant (*p* = 0.384).

### 3.2 Interventions to Control Epistaxis

Epistaxis was controlled at the emergency department visits in different manners: During the pre‐sclerotherapy period, bipolar cauterization was utilised 12 times and non‐absorbable nasal packing 2 times. During the post‐sclerotherapy period, bipolar cauterization was applied 5 times, silver nitrate cauterization once but non‐absorbable nasal packing was not needed. At planned outpatient visits during the pre‐sclerotherapy period, laser therapy was utilised to prevent epistaxis 58 times, and during the post‐sclerotherapy period 56 times respectively. Arterial embolization was necessary only once during the pre‐sclerotherapy period. No procedures requiring OR were observed. No statistically significant change in different intervention modalities was seen before and after the initiation of polidocanol sclerotherapy. Medical treatments were not examined in this study.

## 4 Discussion

Treating epistaxis in HHT patients is demanding and no clear consensus exists on the best method. Here, we study for the first time the effect of polidocanol sclerotherapy on epistaxis‐related visits in HHT patients. Our study did not demonstrate significant changes in the quantities of emergency department visits, outpatient visits, or different procedures before and after the initiation of polidocanol sclerotherapy. However, the current international HHT guideline as well as previous research and clinical experience supports the use of ablative therapies such as sclerotherapy in the management of HHT‐related epistaxis when conservative methods are insufficient [1].

The advantage of polidocanol sclerotherapy is its feasibility for outpatient settings under local anaesthesia and its good tolerability. Polidocanol sclerotherapy is considered cost‐effective as no OR nor general anaesthesia is usually required. Previous studies have shown that polidocanol reduces the frequency and severity of epistaxis in HHT patients, as well as improve their quality of life. In the first major study on polidocanol sclerotherapy for HHT‐related epistaxis by Morais et al., nosebleed frequency and severity decreased in 95% of the 45 cases without significant side effects [8]. In Marcos et al.'s study, polidocanol sclerotherapy significantly reduced the frequency and severity of nosebleeds evaluated by ESS [7]. Both studies also demonstrated improvements in quality of life. A recent systematic review showed positive yet limited data on the efficacy of sclerotherapy for HHT‐related epistaxis [10]. All studies reported improvement in epistaxis, but the lack of uniform reporting measures precluded the analysis. However, these studies did not report effects on the emergency visits.

This is the first study that investigates the impact of polidocanol sclerotherapy on epistaxis‐related emergency visits in HHT patients. These visits and emergency OR procedures can put a significant strain on our health care system, and therefore it is an important factor when assessing treatment efficacy. Overall, emergency department visits and outpatient visits in our study somewhat increased but neither result was statistically significant. Therefore, the reduction in nosebleed severity and frequency demonstrated in previous studies did not clearly reflect in our results. However, our clinical observation is that many HHT patients tend to struggle with nosebleeds without seeking medical help. After initiating sclerotherapy with a continuous care relationship, HHT patients may have sought help more easily also in acute epistaxis. Moreover, HHT‐related epistaxis tends to aggravate with age, which may affect our results. We also found that bipolar cauterization was mainly utilised to control epistaxis at emergency settings. However, it should be avoided in HHT patients because it can stimulate the formation of novel telangiectasia. This finding calls for more awareness among emergency department doctors in treating HHT‐related epistaxis.

Our study has some limitations. First, our patient number was relatively low, and many HHT patients were excluded due to insufficient follow‐up data. Second, due to technical reasons, we did not have access to epistaxis‐related primary health care emergency visits of our HHT cohort. Due to the retrospective study setting, sufficient ESS data was unavailable, although such evaluation would have been beneficial for a more comprehensive assessment of treatment outcomes. Based on our clinical experience, HHT patients are generally satisfied with polidocanol sclerotherapy. In future studies, standardised tools such as the ESS and the quality‐of‐life questionnaires will help to analyse the treatment efficacy.

To determine the impact of polidocanol sclerotherapy on the epistaxis‐related emergency department visits, larger studies and longer follow‐up are needed. HHT‐related epistaxis in the emergency setting is always demanding to treat and therefore it is crucial to find the best methods to prevent these emergency situations.

## Author Contributions

J.W. and E.C. designed the work; E.‐L.K., J.W., E.C. acquired and analysed data; E.‐L.K., J.W., E.C. drafted, revised and approved the manuscript; E.‐L.K., J.W., E.C. agree to be accountable for all aspects of the work.

## Ethics Statement

This retrospective study was approved by the Ethics Committee of Helsinki University Hospital.

## Consent

Informed consent was not required as the study used anonymized data from existing medical records. All personal identifiers were removed to maintain participant confidentiality, and data were securely stored in compliance with institutional data protection protocols. This research adhered to ethical guidelines set forth by the Declaration of Helsinki. The study posed minimal risk to participants, as the data were collected for clinical purposes, and all efforts were made to protect privacy and confidentiality.

## Conflicts of Interest

The authors declare no conflicts of interest.

## Data Availability

The data that support the findings of this study are available on request from the corresponding author. The data are not publicly available due to privacy or ethical restrictions.
